# Dynamic immune state transitions and metastatic niche remodelling drive osteosarcoma evolution as a barrier-restricted immune-cold ecosystem

**DOI:** 10.3389/fimmu.2026.1919497

**Published:** 2026-08-24

**Authors:** Jiangyou Shi, Rongchun Chen

**Affiliations:** Department of Spine Surgery, Ganzhou Hospital-Nanfang Hospital, Southern Medical University (Ganzhou People’s Hospital), Ganzhou, China

**Keywords:** immune-cold tumour, immunotherapy, metastatic niches, osteosarcoma, tumour microenvironment

## Abstract

Osteosarcoma exhibits limited responsiveness to immune checkpoint blockade, a feature commonly referred to as immune coldness. However, interpreting immune coldness as a fixed tumour trait may underestimate the spatially and temporally dynamic processes that govern immune resistance. Accumulating evidence instead supports an evolutionary ecosystem view, in which tumour-intrinsic defects in immune recognition, lymphocyte exclusion, myeloid predominance, and metastatic niche formation collectively contribute to clinically non-productive immunity, rather than representing discrete or mutually exclusive stages. Single-cell, spatial, clinical, and translational studies further highlight pronounced heterogeneity among primary, treatment-exposed, and metastatic lesions, with particularly distinct immune architectures observed in pulmonary sites. In this context, lung metastases are not merely endpoints of dissemination, but active immune-modulatory niches in which macrophages, granulocytic myeloid-derived suppressor cells, fibroblasts, and spatially restricted lymphocytes jointly shape therapeutic resistance. These observations motivate a shift away from modality-centred therapeutic escalation toward immune state–guided stratification, sequential reprogramming of the tumour ecosystem, and barrier-specific treatment strategies. Separating clinical evidence from patient-derived, translational, and preclinical findings further provides a framework for prioritizing spatial biomarkers, designing adaptive clinical trials, and developing lung-metastasis–focused immunotherapeutic approaches.

## Introduction

Osteosarcoma remains a major therapeutic challenge in the metastatic, recurrent, or refractory setting (1–3). Although immune checkpoint blockade has reshaped treatment paradigms in several malignancies, comparable efficacy has not been observed in osteosarcoma. In the phase II SARC028 trial, pembrolizumab showed activity in selected soft-tissue sarcomas; however, no meaningful signal emerged in the bone sarcoma cohort, with only a single objective response among 22 patients with osteosarcoma (4). Likewise, in the Alliance A091401 study, nivolumab monotherapy produced limited responses in unselected metastatic sarcoma, while dual checkpoint inhibition with nivolumab and ipilimumab yielded only modest overall benefit at the trial level (5). Subsequent combination strategies have continued to attract interest but have not substantially changed this overall pattern (6). Although regimens such as lenvatinib plus pembrolizumab have induced responses in selected sarcoma subtypes, and nivolumab-based combinations remain under investigation, clinical efficacy in osteosarcoma has generally been inconsistent and modest, often constrained by toxicity and variable response rates (7, 8). Collectively, these findings underscore a persistent paradox in osteosarcoma immunotherapy. The central issue is therefore not whether immune-based therapies can be applied in this disease, but rather why durable and productive antitumour immunity rarely emerges despite the availability of multiple immunotherapeutic strategies (9, 10).

Recent reviews have summarized the osteosarcoma tumour microenvironment, immunotherapeutic strategies, and approaches aimed at converting immune-cold tumours into immune-responsive states (11–14). These works have expanded understanding of immune-cell composition, checkpoint signalling, myeloid biology, cellular therapies, oncolytic platforms, nanomedicine, and broader tumour–microenvironment regulation. Nevertheless, most existing frameworks are still organized around discrete cell populations, signalling pathways, or therapeutic modalities. This structure can obscure how immune barriers emerge in a context-dependent manner, interact across spatial and anatomical compartments, and jointly shape therapeutic response. An alternative interpretation views osteosarcoma immune resistance as a dynamic ecological process rather than a collection of independent defects. From this standpoint, osteosarcoma can be conceptualized as an evolving immune ecosystem composed of interconnected immune states that arise during tumour progression, therapeutic exposure, and metastatic dissemination (15). Within this framework, recognition failure, immune exclusion, myeloid predominance, and metastatic niche formation converge to produce therapeutic resistance and non-productive immunity. The latter should therefore be regarded as a downstream clinical manifestation rather than a distinct immune state per se (16). Accordingly, transcriptomic, single-cell, spatial, and metastatic evidence is presented prior to the introduction of a conceptual model. This model is intended to organize testable biological barriers and therapeutic hypotheses, without implying that a fixed or linear biological sequence has been clinically established.

Rather than representing a single biological entity, immune coldness encompasses multiple immune states with distinct underlying mechanisms and therapeutic implications. In osteosarcoma, emerging studies have identified immune-low transcriptomic subtypes, lymphocyte-excluded metastatic lesions, exhausted T-cell programs, macrophage-enriched suppressive niches, and tumour-intrinsic defects in antigen presentation (16–20). Single-cell, spatial, and multi-omics analyses further indicate that interactions among malignant cells, immune populations, stromal compartments, and organ-specific metastatic constraints give rise to regionally distinct immune ecosystems, rather than a uniform absence of immunity (15, 18, 21). This heterogeneity carries important therapeutic implications. Tumours with limited T-cell recruitment, spatial T-cell exclusion, T-cell dysfunction, or impaired MHC-I-mediated antigen presentation may all fall under the umbrella of immune-cold disease, yet each is driven by fundamentally different biological constraints. As a result, similar immune phenotypes may require markedly different therapeutic strategies, underscoring the need to view immune coldness as a dynamic and heterogeneous spectrum of immune states rather than a single pathological condition.

The following sections integrate clinical, spatial, single-cell, and mechanistic evidence to examine how immune barriers emerge and evolve during osteosarcoma progression and pulmonary metastasis. This framework supports a shift from empiric treatment intensification toward immune state–guided stratification, barrier-matched intervention, and sequential reprogramming.

## Refined immune state taxonomy

**Figure 1:**
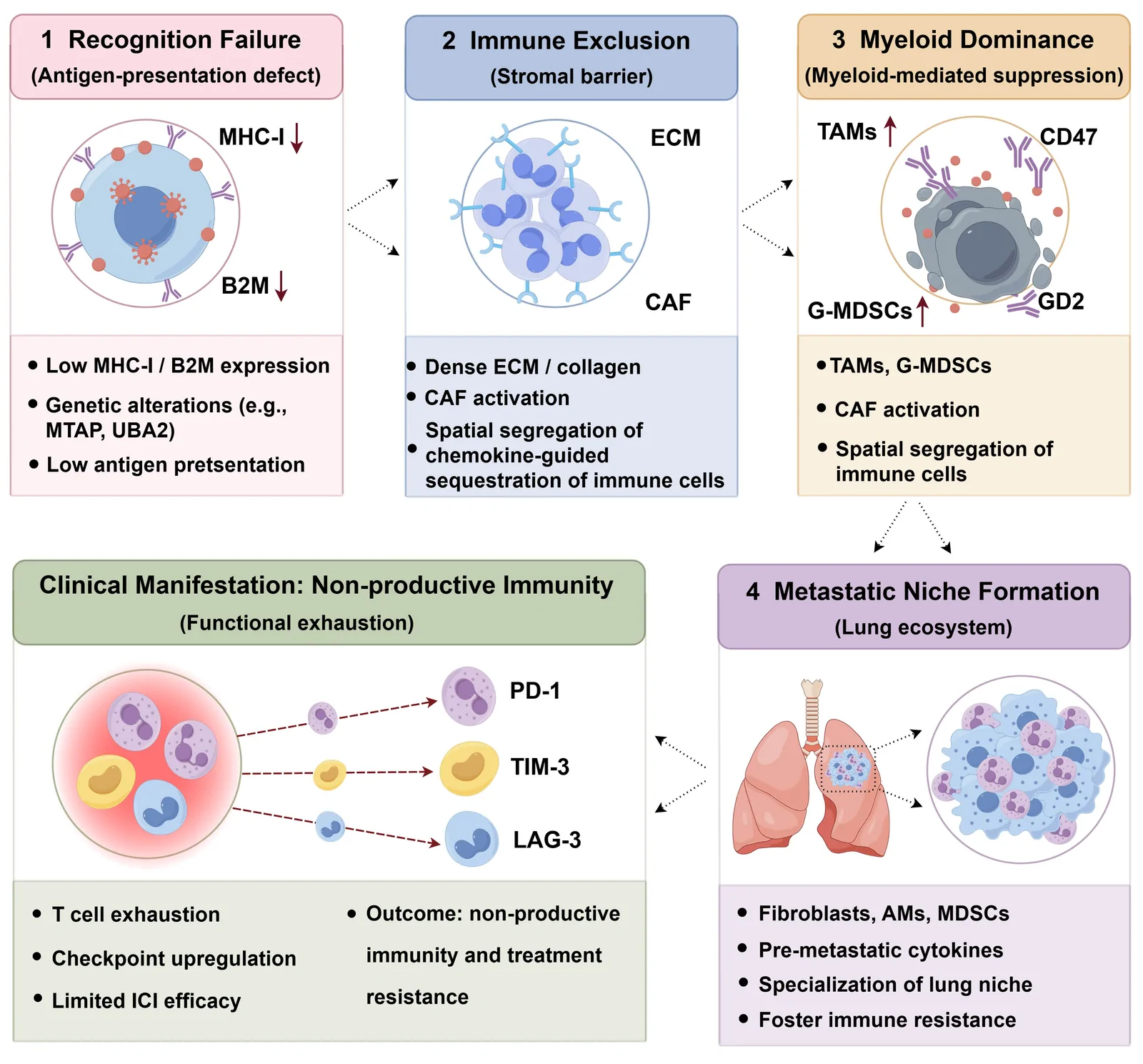
Operational immune state barriers in osteosarcoma immune coldness. This framework conceptualizes osteosarcoma immune coldness as four interconnected barriers rather than fixed clinical stages. Recognition failure includes reduced major histocompatibility complex class I (MHC-I) or beta-2-microglobulin (B2M) expression, antigen-presentation impairment and context-specific metabolic constraints. Immune exclusion describes lesions in which T cells or inflammatory signals are present but remain spatially segregated from tumour-cell regions by extracellular matrix (ECM), collagen, cancer-associated fibroblast (CAF) programs, vascular or lymphatic architecture, macrophage-rich fibrotic niches, or compartmentalized chemokine gradients that retain or redirect effector lymphocytes away from malignant-cell compartments. Myeloid dominance reflects enrichment of tumour-associated macrophages (TAMs), granulocytic myeloid-derived suppressor cells (G-MDSCs) and phagocytosis-checkpoint pathways such as CD47, MerTK and GD2-SIGLEC. Metastatic-niche formation emphasizes lung-specific fibroblast, macrophage and MDSC programs that condition premetastatic and established metastatic sites. Non-productive immunity is shown as the downstream clinical and treatment-failure phenotype caused by unresolved barriers, not as an independent fifth ecological stage.

Current evidence can be organized into four operational immune state barriers: recognition, access, suppression, and organ-specific metastatic adaptation (**Figure 1**). The first category, intrinsically low-immunogenic disease, is characterized by impaired immune recognition and priming, including immune-low transcriptomic subtypes, reduced inferred immune-cell abundance, MTAP-associated methionine dependency, hypoxia- and lactate-driven immunosuppression, and tumour-intrinsic defects in antigen presentation (17, 20, 22–24). The second, immune-excluded disease, is defined by immune activity that remains spatially separated from tumour-cell compartments. In paediatric pulmonary metastases, this is reflected by peripheral inflammatory signalling, fibrocollagenous encapsulation, lymphocyte exclusion, and peritumoral macrophage accumulation, while spatial multiplex immunofluorescence further links PD-1-positive T cells and myeloid-derived suppressor cells to specific survival-associated cellular neighbourhoods (18, 21). The third category, myeloid–stromal-dominated disease, describes an actively suppressive ecosystem in which immune cells are present but functionally constrained by their organization and context. Underlying mechanisms include CCL2-driven macrophage recruitment, GD2-associated impairment of phagocytosis, CD47- and MerTK-related inhibitory signalling, and cancer-associated fibroblast programs (19, 25–28). Finally, metastatic-niche–remodelled disease highlights the organ-specific dimension of immune coldness. Osteosarcoma cells establish permissive pulmonary niches through CXCL14-mediated activation of integrin α11β1-positive fibroblasts, as well as premetastatic conditioning via extracellular-vesicle–associated S100A11 that recruits lung interstitial macrophages and granulocytic myeloid-derived suppressor cells (29, 30). Together, these states suggest that immune coldness in osteosarcoma is spatially and temporally dynamic rather than a fixed property of the primary tumour (**Figure 1**).

Collectively, these immune state barriers highlight that osteosarcoma immune coldness arises from distinct but interconnected mechanisms affecting immune recognition, spatial access, and suppressive microenvironments. Rather than representing a single static phenotype, these states reflect a dynamic and context-dependent spectrum shaped by tumour evolution and organ-specific metastatic adaptation.

## Immune state transition revealed by single-cell and spatial omics

**Figure 2:**
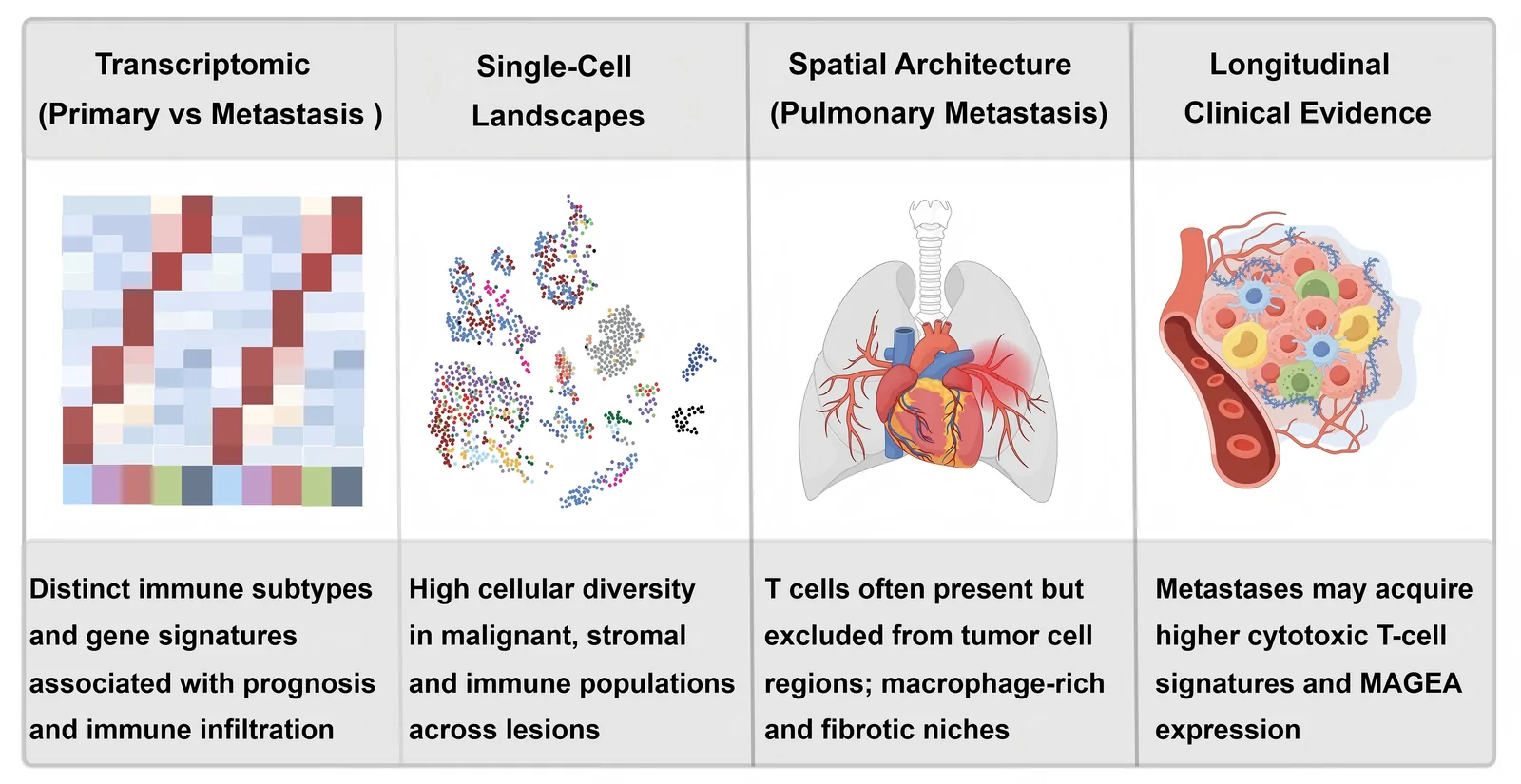
Evidence layers supporting dynamic immune state transitions. The schematic summarizes the complementary evidence base used in this Review. Transcriptomic studies distinguish immune-high and immune-low subtypes and immune-related gene signatures; single-cell analyses reveal malignant, stromal and immune-cell diversity, macrophage remodelling and exhausted or regulatory T-cell states; spatial profiling of pulmonary metastases demonstrates fibrocollagenous and macrophage-rich compartments in which T cells may be present but excluded from tumour-cell regions; and longitudinal patient-sample analyses indicate that metastases may acquire stronger cytotoxic T-cell signatures, MAGEA expression or altered checkpoint status. This figure is a synthesis of available evidence rather than proof of a single validated trajectory.

Single-cell and spatial technologies provide some of the most compelling evidence that immune coldness is not a static phenotype but an evolving ecosystem state. While bulk immune profiling captures population-level heterogeneity, it cannot resolve whether immune cells physically engage tumour cells, remain restricted to stromal compartments, or assemble within suppressive cellular neighbourhoods (**Figure 2**). In contrast, single-cell RNA sequencing, spatial transcriptomics, and multiplex imaging enable direct mapping of immune organization across primary tumours, treatment-exposed lesions, recurrent disease, and pulmonary metastases (31, 32). Evidence indicates that immune heterogeneity is already present at early disease stages. Single-cell analyses of advanced osteosarcoma have revealed diverse malignant, stromal, and immune-cell populations across primary, recurrent, and metastatic lesions, with prominent macrophage remodelling in pulmonary metastases (15). Similarly, treatment-naive tumours already exhibit substantial microenvironmental complexity, suggesting that immune coldness is not solely a consequence of therapeutic pressure but is embedded within tumour development from its earliest phases (33).

Immune states further evolve throughout disease progression and treatment exposure. Although a complete longitudinal atlas is still lacking, emerging single-cell and immunogenomic studies indicate substantial plasticity within osteosarcoma immune ecosystems (**Figure 2**). T-cell exhaustion programs and heterogeneous immune-response states have been identified through single-cell transcriptomic profiling (16), whereas serial-sample analyses have reported enrichment of cytotoxic T-cell–associated signatures and increased antigenicity in metastatic lesions compared with matched primary tumours (34). These findings challenge a strictly linear progression toward immune coldness and instead support branching evolutionary trajectories in which immune activation and immune resistance may coexist. Pulmonary metastasis provides a particularly clear example of this complexity. Spatial profiling has revealed peripheral inflammatory signalling, fibrocollagenous encapsulation, lymphocyte exclusion, and macrophage accumulation within paediatric lung metastases (18). Complementary spatial imaging studies have further identified survival-associated cellular neighbourhoods characterized by interactions between PD-1–positive T cells and myeloid-derived suppressor cells (21). Together, these observations suggest that immune coldness can shift from immune scarcity in one context to spatially confined or functionally suppressed immunity in another.

These data support a transition-based view of immune state organization rather than static immune classification. Future studies should integrate tissue origin, treatment history, metastatic site, and profiling modality into a unified analytical framework, recognizing that primary tumours, therapy-exposed specimens, recurrent lesions, and pulmonary metastases represent distinct immunological contexts rather than interchangeable snapshots of disease. Collectively, these evidence layers provide the empirical foundation for the conceptual framework presented in the following section (**Figure 2**).

## A conceptual framework for immune state evolution in osteosarcoma

**Figure 3:**
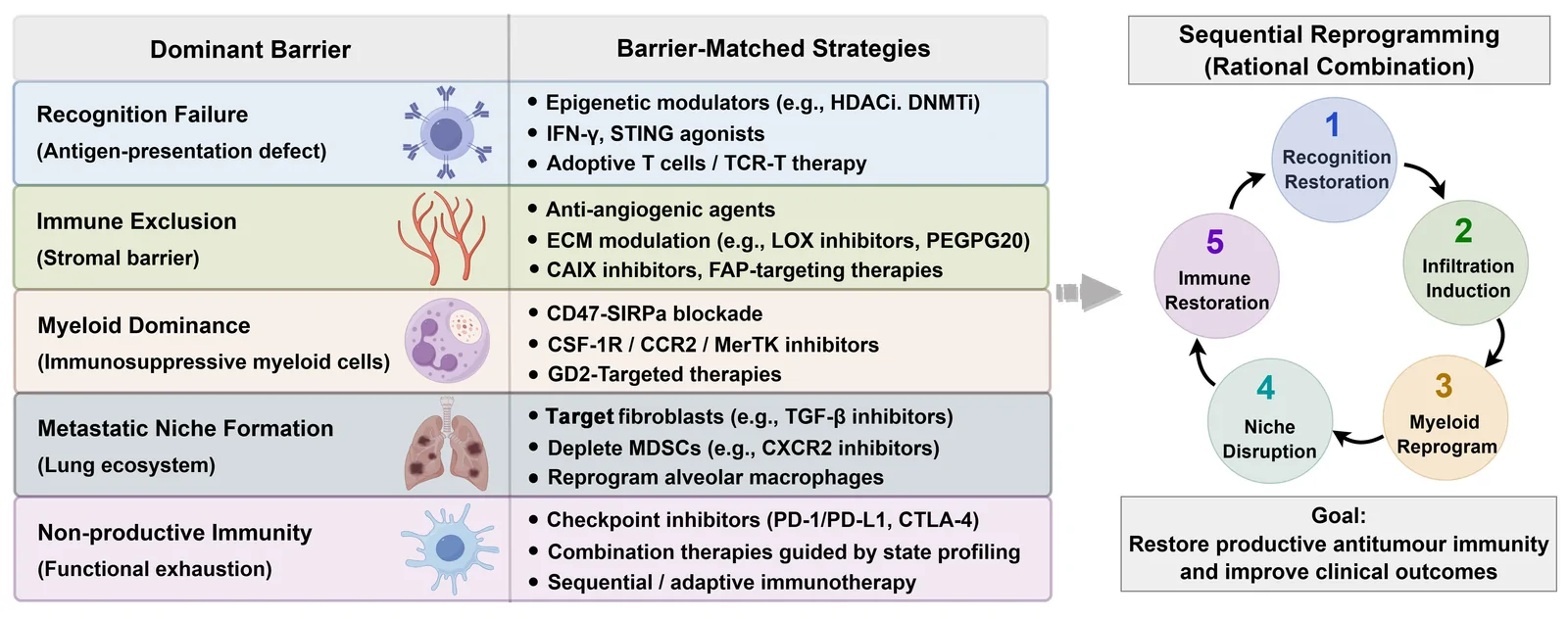
Barrier-matched therapeutic logic for sequential reprogramming. This schematic links dominant immune barriers to therapeutic classes that could be tested with prespecified pharmacodynamic objectives. Recognition failure may require antigen-presentation restoration or context-dependent priming before checkpoint blockade; immune exclusion may require extracellular-matrix, vascular or CAF/fibrosis-directed reprogramming before T-cell entry can be expected; myeloid dominance may require macrophage, MDSC or phagocytosis-checkpoint modulation; and lung metastatic niches may require site-specific disruption of fibroblast, wound-healing and innate-immune programs. Non-productive immunity is represented as a downstream treatment-failure phenotype. The circular sequence illustrates a hypothesis-generating strategy, not a clinically validated order of therapy.

Although no unified model currently exists to explain the emergence of immune coldness in osteosarcoma, multiple studies have independently described immune-low states, defects in antigen presentation, spatial immune exclusion, myeloid suppression, and metastatic niche formation (35, 36). Integrating these observations, we propose a conceptual framework in which immune resistance is organized into interconnected immune states rather than isolated mechanisms. Within this framework (**Figure 3**), Phase 1 corresponds to recognition failure, encompassing immune-low transcriptomic phenotypes, metabolic constraints on immune responsiveness, and tumour-intrinsic loss of MHC-I-mediated antigen presentation (17, 20, 23). Phase 2 reflects immune exclusion, in which lymphocytes or inflammatory signals are present but remain spatially segregated from tumour-cell regions by fibrocollagenous, stromal, or regional barriers (18, 21). Phase 3 denotes myeloid-dominant disease, characterized by macrophage states, myeloid-derived suppressor cells, and phagocytosis-related checkpoints that collectively establish a suppressive ecosystem; representative mechanisms include MerTK-mediated efferocytosis, CD47-associated phagocytosis resistance, and GD2–SIGLEC–mediated impairment of macrophage function (15, 19, 26, 27). Phase 4 represents metastatic immune-ecosystem formation, particularly in the lung, where tumour-derived signals promote fibroblast activation, extracellular matrix remodelling, recruitment of macrophages and granulocytic myeloid-derived suppressor cells, and establishment of a site-specific niche (29, 30, 37, 38). Rather than constituting a distinct fifth immune ecosystem, non-productive immunity is used here to describe the downstream clinical manifestation of unresolved recognition, spatial, myeloid, metabolic, and effector-level barriers. The limited activity observed in SARC028 and Alliance A091401 reflects this clinical phenotype at the trial level, although these studies do not resolve which upstream barrier predominates in individual osteosarcoma lesions (4, 5).

Importantly, these states should not be interpreted as strictly sequential (**Figure 3**). Osteosarcoma is genetically, spatially, and temporally heterogeneous, and multiple immune states may coexist within the same patient or even within a single metastatic lesion. Human evidence supports a branching rather than linear trajectory. In a cohort of 30 longitudinal samples from seven patients, immunogenomic and imaging-mass-cytometry analyses revealed increased cytotoxic T-cell signatures and elevated MAGEA cancer-testis-antigen expression in metastatic lesions compared with primary tumours (34). Spatial profiling further identified areas of central T-cell infiltration in selected metastases. Together, these findings argue against a universal cold-to-colder progression and instead support the coexistence of partially inflamed yet therapeutically resistant states in which antigenicity and immune activation fail to translate into durable tumour control. This framework is therefore intended as diagnostic rather than deterministic, asking whether the dominant barrier at a given tissue site and treatment stage lies in failed recognition, spatial exclusion, myeloid suppression, metabolic constraint, or lung-specific niche adaptation (**Figure 3**). These mechanisms are not interchangeable, and their separation provides a rationale for barrier-matched combinations and sequential reprogramming strategies, although their optimal ordering remains hypothesis-generating until validated in biomarker-driven clinical studies (39, 40).

Overall, this framework reframes immune coldness in osteosarcoma as a dynamic and heterogeneous ecosystem rather than a fixed tumour attribute. It provides a structured basis for linking spatial and molecular heterogeneity to therapeutic resistance in a barrier-specific manner. Ultimately, its value lies in generating testable hypotheses for stratified and sequential immunotherapeutic intervention.

## Why T cells fail in osteosarcoma

Despite the diversity of immune-cold states observed in osteosarcoma, many ultimately converge on a shared endpoint: failure of effective T-cell–mediated tumour control. This failure can arise at multiple stages of the antitumour immune response, including impaired immune recognition, restricted tumour access, and sustained functional suppression following tumour engagement. Identifying where this process is interrupted is important, as distinct barriers are unlikely to respond to identical therapeutic strategies. The most upstream barrier is recognition failure. UBA2-high osteosarcoma has been associated with autophagy-mediated degradation of surface MHC-I, reduced CD8^+^ T-cell infiltration, and increased sensitivity to checkpoint blockade following UBA2 inhibition *in vivo* (20). These findings suggest that defects in antigen presentation may render tumour cells effectively invisible to cytotoxic lymphocytes. In this context, checkpoint blockade alone is unlikely to restore immunity, as the initiating step of immune recognition has not been established. A second barrier is immune exclusion. Spatial profiling studies indicate that lymphocytes may remain physically separated from tumour-cell compartments by fibrocollagenous encapsulation and a suppressive peritumoral architecture (18). As a result, the presence of immune cells does not necessarily reflect functional tumour surveillance, particularly when effector cells fail to reach tumour-cell–proximal niches. Chemokine organization may provide an additional layer of immune exclusion. In other solid tumours, extracellular-matrix architecture, activated fibroblasts, and spatially compartmentalized chemokine signals can retain or redirect effector lymphocytes towards stromal, perivascular, or lymphatic niches rather than tumour-cell compartments (41–43). The CXCL12–CXCR4 axis provides a representative example: CXCL12 produced by activated fibroblasts can sequester CD8^+^ T cells within stromal regions, whereas lymphatic CXCL12 can promote effector T-cell egress from tumours (42, 44, 45). In osteosarcoma pulmonary metastases, spatial profiling identified fibrocollagenous encapsulation, lymphocyte exclusion, and spatially heterogeneous CXCR4-associated signalling across intratumoral and extratumoral compartments (18). However, direct evidence that these gradients actively misdirect effector lymphocytes in osteosarcoma remains limited. Chemokine-guided sequestration is therefore best positioned within the immune-exclusion barrier, acting alongside fibrocollagenous, vascular, lymphatic and CAF-associated constraints rather than constituting a separate immune state (41–45). A third barrier involves T-cell dysfunction and active suppression. Single-cell transcriptomic analyses have identified exhausted T-cell states and heterogeneous immune-response programs in osteosarcoma (16), while spatial multiplex imaging has revealed cellular neighbourhoods enriched for PD-1–positive T cells and myeloid-derived suppressor cells in metastatic lesions (21). Together, these observations indicate that T-cell dysfunction may persist even after successful recruitment and tumour infiltration, driven by inhibitory receptor signalling and suppressive microenvironmental interactions.

Beyond these canonical barriers, regulatory immune programs likely represent an additional and less well-defined layer of T-cell impairment. Although evidence for tertiary lymphoid structures in osteosarcoma remains limited, emerging single-cell analyses have described regulatory T-cell populations characterized by oxidative phosphorylation, angiogenic activity, and mTORC1-associated signalling, alongside predicted interactions involving CXCL12–CXCR4 and TGFB1 pathways. Independent studies have also reported regulatory dendritic-cell states capable of promoting Treg recruitment, together with tumour-cell MHC-I downregulation and CD24-mediated macrophage evasion mechanisms (46). Collectively, these findings extend the concept of T-cell failure beyond exhaustion alone, encompassing absence, exclusion, dysfunction, and active suppression as distinct biological states with potentially different therapeutic implications. In contrast, robust osteosarcoma-specific evidence for mature tertiary lymphoid structures, their spatial organization, and their predictive value for immunotherapy response remains limited. Defining these regulatory architectures therefore represents an important direction for future immune state classification and biomarker development.

Overall, these observations support a view of T-cell failure in osteosarcoma as a multistage and context-dependent process, rather than a single uniform defect. Distinct barriers—including impaired recognition, spatial exclusion, and functional suppression—may dominate in different lesions or disease contexts. This heterogeneity underscores the need for barrier-aware immune state classification to guide therapeutic strategies.

## The lung as an immune-reprogramming organ

Lung metastasis should be viewed not only as the terminal outcome of tumour dissemination, but as a site-specific immune ecosystem that actively reshapes osteosarcoma immunity both before and after metastatic colonization (31). This distinction is particularly relevant given that pulmonary metastasis remains the leading cause of mortality in osteosarcoma, whereas immune profiling of primary tumours may not capture the barriers that ultimately determine metastatic persistence and therapeutic resistance. Evidence increasingly indicates that immune reprogramming begins prior to overt tumour-cell arrival. Premetastatic niche formation provides a mechanistic link between primary-tumour signalling and lung immune ecology. Osteosarcoma-derived extracellular vesicles carrying S100A11 activate lung interstitial macrophages and recruit granulocytic myeloid-derived suppressor cells via CXCL2 signalling, thereby establishing a permissive premetastatic environment (30). In parallel, tumour-derived CCL2 promotes macrophage accumulation and supports pulmonary metastatic dissemination (25). These findings suggest that the lung can undergo immunological conditioning before detectable metastatic outgrowth. Beyond myeloid recruitment, tumour-derived factors have also been associated with airway injury, inflammatory activation, neutrophil infiltration, fibroblast activation, and extracellular-matrix deposition prior to tumour cell colonization (37). In this context, EFEMP1 has been implicated as a key mediator, with higher expression correlating with adverse clinical outcomes. Although the causal sequence has been primarily defined in experimental models, these observations collectively implicate neutrophil-associated inflammation and matrix remodelling as early components of lung-specific immune reprogramming.

Following metastatic seeding, stromal and myeloid compartments become central regulators of immune access and function. Metastatic osteosarcoma cells recruit M2-like tumour-associated macrophages through CCL2 signalling, while pharmacological or antibody-mediated blockade of this axis suppresses pulmonary metastasis without affecting primary tumour growth (25). In addition, stem-like tumour cells cooperate with fibroblasts through the CXCL14–integrin α11β1 pathway to establish a supportive metastatic niche, and disruption of this interaction enhances antitumour immunity and reduces metastatic burden (29). Spatial profiling of pulmonary metastases further reveals fibrocollagenous encapsulation, lymphocyte exclusion, and peritumoral macrophage accumulation (18), indicating that lung metastases develop a distinct spatial architecture of immune resistance. Emerging data also suggest that metastatic niches may resemble non-resolving tissue repair programs. Integrated single-cell and spatial analyses in murine models and patient-derived samples have identified acute alveolar epithelial injury, profibrotic epithelial intermediates, macrophage accumulation, and extracellular-matrix deposition surrounding disseminated osteosarcoma cells (38). Pharmacological inhibition of this fibrosis-associated program using nintedanib reduced metastatic progression in both syngeneic and xenograft models. Although these findings remain preclinical, they implicate fibrosis and tissue-repair signalling as potentially targetable components of lung-specific immune exclusion.

Myeloid populations provide an additional axis linking metastatic progression, immune suppression, and therapeutic resistance. Granulocytic myeloid-derived suppressor cells, macrophages, and neutrophil-associated programs are increasingly recognized as central mediators of metastatic immune dysfunction. Tumour-derived G-CSF establishes an immunosuppressive milieu that impairs GD2 CAR-T-cell efficacy in osteosarcoma models (47). Similarly, GD2–SIGLEC interactions suppress macrophage phagocytosis and facilitate pulmonary metastatic progression (19). Collectively, these observations support the view that lung metastasis functions not merely as an anatomical endpoint of dissemination, but as an active immune-reprogramming organ that continuously shapes metastatic immune states. Comparative canine–human studies further reinforce the existence of conserved lung-metastatic immune ecosystems. Cross-species analyses have identified shared tumour–microenvironment subtypes in canine and human osteosarcoma (48). In spontaneous canine metastatic disease, losartan reduced tumour-associated monocyte recruitment and showed clinical benefit when combined with toceranib (49). Although species-specific differences must be considered, canine models may provide a useful platform for prioritizing conserved immune barriers and identifying pharmacodynamic biomarkers for lung-metastasis-focused therapeutic strategies.

Overall, lung metastasis represents an active and evolving immune ecosystem that shapes osteosarcoma immunity through both premetastatic conditioning and post-seeding niche remodelling. Immune suppression is driven by coordinated stromal, myeloid, and fibrotic programs that establish spatially organized resistance rather than a uniform loss of immunity. These findings highlight lung metastasis as a dynamic immunological compartment and a key driver of therapeutic resistance in osteosarcoma.

## Tumour-intrinsic and myeloid-stromal drivers of transition

Tumour-intrinsic programs can establish immune coldness even before the surrounding microenvironment is fully assembled. In osteosarcoma, these programs encompass regulation of antigen presentation, induction of checkpoint ligands, metabolic dependencies, and secretion of niche-modulating factors. For instance, GBP1 promotes PD-L1 expression via CDK9 activation and STAT3 phosphorylation (50), suggesting that checkpoint regulation can originate within tumour cells. However, PD-L1 expression alone does not reliably predict response, as unselected checkpoint blockade trials in osteosarcoma have shown limited efficacy (4, 8). Rather than functioning as a predictive marker, PD-L1-associated pathways may identify tumours in which checkpoint signalling is present but overridden by upstream constraints such as defective immune recognition, myeloid suppression, or spatial exclusion. Metabolic rewiring represents another axis of immune modulation. MTAP-deleted osteosarcomas depend on methionine metabolism and display immune-cold microenvironments associated with checkpoint resistance (23). In preclinical models, methionine supplementation increased PD-L1 expression, activated immune-related signalling, and enhanced responsiveness to checkpoint blockade, indicating that genetic lesions can shape partially reversible immune states.

Beyond tumour-cell metabolism, emerging evidence suggests that microbially derived metabolites may also contribute to immune modulation. Integrated analyses of 16S rRNA, circulating microbial DNA, bulk, and single-cell datasets have revealed altered microbial composition and butyrate-associated metabolic programs in metastatic osteosarcoma (51). Functionally, butyrate promotes tumour-cell migration through Wnt–β-catenin signalling and accelerates metastatic progression *in vivo*, although these observations remain preliminary. Together, these findings extend metabolic immune regulation to include microbiome-derived signals that may influence metastatic competence. Myeloid and stromal compartments further consolidate immune coldness at the ecosystem level. MerTK-mediated efferocytosis promotes immune tolerance, M2 polarization, and PD-L1 expression in osteosarcoma models (27). CD47 expression correlates with metastatic disease at diagnosis and inhibits macrophage-mediated phagocytosis (26), while GD2 engagement of SIGLEC-family receptors impairs macrophage function and facilitates pulmonary metastasis (19). Cancer-associated fibroblasts expressing CD36 have also been associated with resistance to anti-PD-1 therapy and may represent a targetable component of the suppressive niche (28). Collectively, these processes suggest that macrophages and fibroblasts function not merely as passive responders but as active regulators of immune state stability, integrating tumour-intrinsic and microenvironmental signals to sustain immune coldness and shape therapeutic resistance. Earlier mechanistic studies further support this model. Osteosarcoma-derived exosomal ELFN1-AS1 promotes M2 macrophage polarization via miR-138-5p/miR-1291–CREB1 signalling, and in turn enhances tumour progression (52).

Collectively, tumour-intrinsic programs and downstream stromal, metabolic, and microbial interactions cooperate to establish and stabilize immune coldness in osteosarcoma before and during microenvironmental assembly. These interconnected processes position immune suppression as an ecosystem-level property emerging from continuous crosstalk between tumour cells and their surrounding biological context rather than a single pathway-driven event.

## Why immunotherapy has underperformed in osteosarcoma

Immunotherapy in osteosarcoma has largely underperformed because most strategies have not been aligned with dominant immune barriers. Checkpoint blockade illustrates this limitation. Pembrolizumab demonstrated minimal activity in osteosarcoma within the SARC028 trial, while nivolumab monotherapy showed limited efficacy in unselected metastatic sarcoma cohorts (4, 5). Although combination regimens have generated early signals of activity, osteosarcoma-specific benefit remains uncertain and requires careful subgroup interpretation (7, 8). Several of these limitations arise upstream of PD-1/PD-L1 signalling. Reduced MHC-I antigen presentation restricts the ability of checkpoint blockade to restore immune recognition (20), while stromal exclusion can prevent effector T cells from reaching tumour-cell compartments (18). In addition, macrophage dysfunction mediated by GD2–SIGLEC interactions or CD47-dependent pathways may sustain innate immune suppression even when adaptive checkpoints are pharmacologically engaged (19, 26). Together, these mechanisms suggest that lack of efficacy is not solely attributable to checkpoint resistance, but to failures at multiple non-redundant stages of the immune response. Clinical interpretation is further complicated by cohort heterogeneity. Bone sarcoma trials frequently aggregate osteosarcoma with histologically distinct entities such as chondrosarcoma and Ewing sarcoma (4, 8). Even within osteosarcoma, multiple immune states—including low-immunogenic, immune-excluded, myeloid–stromal-dominated, and metastatic niche–remodelled phenotypes—may coexist across patients and anatomical sites (17, 18, 20). Without stratification by immune state, potentially responsive subgroups may be masked within predominantly non-responsive populations. Mifamurtide illustrates both the potential and the limitations of innate immune activation. In non-metastatic high-grade osteosarcoma, pooled analyses suggested a possible event-free survival benefit when mifamurtide was added to chemotherapy, although multivariable validation remained inconclusive (53). By contrast, in metastatic or recurrent disease, the EuroSarc-Memos trial was terminated early and reported no objective responses among eight patients (54). Correlative analyses have identified immune microenvironment–associated and mifamurtide-linked gene signatures, supporting the hypothesis that therapeutic activity may depend on underlying immune context (55).

Earlier immune-stimulation trials further reinforce the importance of disease stage and treatment adherence. In the EURAMOS-1 good-response cohort, pegylated interferon alfa-2b maintenance following MAP chemotherapy did not significantly improve event-free survival, partly due to delayed initiation and treatment discontinuation (56). Similarly, in the metastatic subset of Intergroup-0133, liposomal MTP-PE showed numerically improved survival outcomes, but the analysis was underpowered and not statistically definitive (57). These studies collectively suggest that cytokine-based immune activation cannot be assumed to exert uniform benefit across distinct immune contexts. Cellular therapies face comparable ecological constraints. Engineering strategies such as CXCR5 or IL-7 co-expression have improved CAR-T efficacy in preclinical osteosarcoma models, whereas tumour-derived G-CSF can suppress CAR-T activity by shaping an immunosuppressive myeloid environment (47, 58). These findings indicate that antigen targeting alone is insufficient unless trafficking, persistence, and myeloid resistance are simultaneously addressed. Early-phase clinical studies further highlight both feasibility and persistent efficacy limitations. In a phase I/II trial of HER2-directed CAR-T cells involving 16 osteosarcoma patients, treatment was well tolerated and demonstrated tumour trafficking and persistence, yet objective responses were rare, with only four of 17 evaluable sarcoma patients achieving stable disease (59). Similarly, dendritic-cell vaccination using autologous tumour lysate was safe in relapsed osteosarcoma, but induced tumour-specific T-cell responses in only a small subset of patients without clear clinical benefit (60). More recent B7-H3 CAR-T approaches have incorporated CXCR2 or CXCR6 to enhance chemokine-guided homing, improving persistence and tumour control in metastatic models (61). Collectively, these observations reinforce that antigen selection alone is insufficient, and must be integrated with strategies that address trafficking, expansion, persistence, and immune suppression within the tumour microenvironment. Classic GD2-directed studies anticipated these constraints. Although GD2 was widely expressed in osteosarcoma specimens, GD2 CAR-T efficacy was limited in xenograft models unless suppressive myeloid populations were reduced with all-trans retinoic acid (62). Subsequent studies further demonstrated GD2 CAR-T cytotoxicity alongside adaptive resistance mechanisms, including PD-L1 upregulation, CAR-T PD-1 expression, and apoptosis, with sub-toxic doxorubicin enhancing tumour killing (63).

These findings indicate that immunotherapy failure in osteosarcoma reflects mismatches between therapeutic mechanisms and multiple upstream immune barriers, including defective antigen presentation, spatial exclusion, and myeloid-mediated suppression. Across checkpoint blockade, cytokine-based approaches, and cellular therapies, durable efficacy appears to require coordinated restoration of antigen recognition, immune trafficking, and effector function within a highly heterogeneous tumour–immune ecosystem.

## Sequential therapeutic reprogramming in osteosarcoma

Therapeutic reprogramming should be evaluated according to both the immune barrier being targeted and the level of supporting evidence. Level I evidence is derived from clinical studies, including checkpoint inhibitor trials and mifamurtide-based regimens. To date, these studies have demonstrated limited activity in unselected populations or context-dependent benefit rather than broadly effective immunotherapy (4, 5, 53, 54). Level II evidence includes translational analyses based on patient samples—such as spatial, single-cell, and correlative studies—that define actionable immune barriers but have not yet established clinical efficacy (18, 21, 55). Level III evidence consists of preclinical or hypothesis-generating approaches, including cellular therapies, nanoparticles, engineered bacteria, metabolic interventions, and stromal reprogramming strategies (23, 47, 58, 64). A central concept emerging from these studies is that effective therapy may require sequential reprogramming of immune barriers rather than single-step intervention. Tumours with defective antigen recognition may require restoration of antigen presentation prior to checkpoint blockade. For example, UBA2 inhibition reduced MHC-I degradation and sensitized osteosarcoma models to PD-1 blockade (20), while MTAP-deficient tumours may depend on metabolic priming, as methionine supplementation enhanced immune signalling and improved checkpoint responsiveness in this context (23). In such settings, restoration of antigen recognition precedes T-cell recruitment and checkpoint engagement. Preclinical studies further support this sequential logic. Etoposide increased MHC-I expression through IL-33–ST2–NF-κB signalling, enhanced CD8^+^ T-cell cytotoxicity, and improved anti–PD-1 efficacy in osteosarcoma models (65). MYC inhibition promoted chemokine expression, strengthened dendritic cell–T cell communication via CD40–CD40L signalling, and sensitized immunocompetent models to PD-1 blockade (66). These findings support pharmacodynamic monitoring of antigen presentation and T-cell recruitment as potential indicators of therapeutic priming, although clinical validation in osteosarcoma remains limited.

Tumours characterized by immune exclusion or myeloid predominance may instead require stromal and myeloid reprogramming prior to checkpoint therapy. STAT3 inhibition modulated macrophage and MDSC activation states and enhanced anti-CD47 activity in lung metastasis models (39, 67). Additional targets include CD47, MerTK, GD2–SIGLEC, and CCL2-associated pathways (19, 25–27). In parallel, stromal interventions such as hydrogel-based delivery systems have been shown to suppress cancer-associated fibroblast activity, induce immunogenic cell death, and improve response to PD-1 blockade in stroma-rich models (40), while targeting OxLDL–CD36 fibroblast signalling further enhances checkpoint efficacy in preclinical settings (28). In this context, stromal and myeloid remodelling is positioned upstream of checkpoint activation. Innate immune activation may be particularly relevant in spatially defined disease contexts such as pulmonary metastasis. In syngeneic lung-metastasis models, particulate β-glucan reduced tumour seeding and established tumour burden through macrophage- and NK-cell–dependent mechanisms, and combination with CD40 agonism further amplified inflammatory responses (68, 69). This represents a form of site-specific innate reprogramming, although it remains preclinical and requires careful safety evaluation before integration with adaptive immunotherapy. Finally, inadequate effector recruitment may require chemokine or innate immune modulation, although translational feasibility remains uncertain. Engineered VNP20009 expressing CCL2 and CXCL9 improved antitumour immunity in lung metastasis models via cGAS–STING–associated immune activation (64), and CXCR5/IL-7 engineering enhanced CAR-T function in osteosarcoma models (58).

Next-generation engineered T-cell platforms could also be incorporated into the barrier-matched paradigm as potential solutions to specific failure modes. In recognition-defective disease, CAR T cells and CD3-engaging bispecific antibodies can redirect T-cell cytotoxicity independently of peptide–MHC presentation, although their efficacy remains dependent on accessible and sufficiently expressed tumour-surface antigens (59, 70). In immune-excluded lesions, chemokine-receptor engineering may improve effector-cell trafficking; for example, CXCR2- or CXCR6-expressing B7-H3 CAR T cells have demonstrated enhanced homing and antitumour activity in preclinical osteosarcoma models (61). In myeloid–stromal-dominated disease, armoured CARs that locally deliver immunostimulatory cytokines or chemokines, or are engineered to resist inhibitory signals, could enhance persistence and remodel suppressive niches, although supporting evidence is largely derived from other solid-tumour models and direct osteosarcoma-specific evidence remains limited (71, 72). For antigen-heterogeneous tumours, switchable or bispecific-engager-secreting CAR platforms, together with T-cell-engaging bispecific antibodies, may broaden target recognition and reduce antigen escape (73, 74). Preclinical osteosarcoma studies using switchable B7-H3 CAR T cells and GD2- or HER2-directed T-cell-engaging bispecific antibodies support the feasibility of these approaches, although safety, antigen density, trafficking and microenvironmental suppression remain important translational constraints (70, 71, 75). However, these approaches are constrained by delivery, trafficking, persistence, antigen heterogeneity, and safety considerations, and should be regarded as experimental reprogramming strategies rather than near-term clinical solutions. Supporting this framework, IL-18 blockade reduced granulocytic and monocytic MDSC accumulation and enhanced anti–PD-1 efficacy in preclinical osteosarcoma models, suggesting that cytokine-driven myeloid recruitment may function upstream of checkpoint resistance (75).

Collectively, these studies support a model in which effective immunotherapy in osteosarcoma requires stepwise and context-dependent reprogramming of distinct immune barriers, rather than uniform single-agent intervention. However, most evidence remains preclinical or correlative, and the clinical feasibility of sequential barrier modulation across antigen presentation, stromal organization, and myeloid suppression remains to be established.

## Barrier-matched clinical trial framework for osteosarcoma

**Figure 4:**
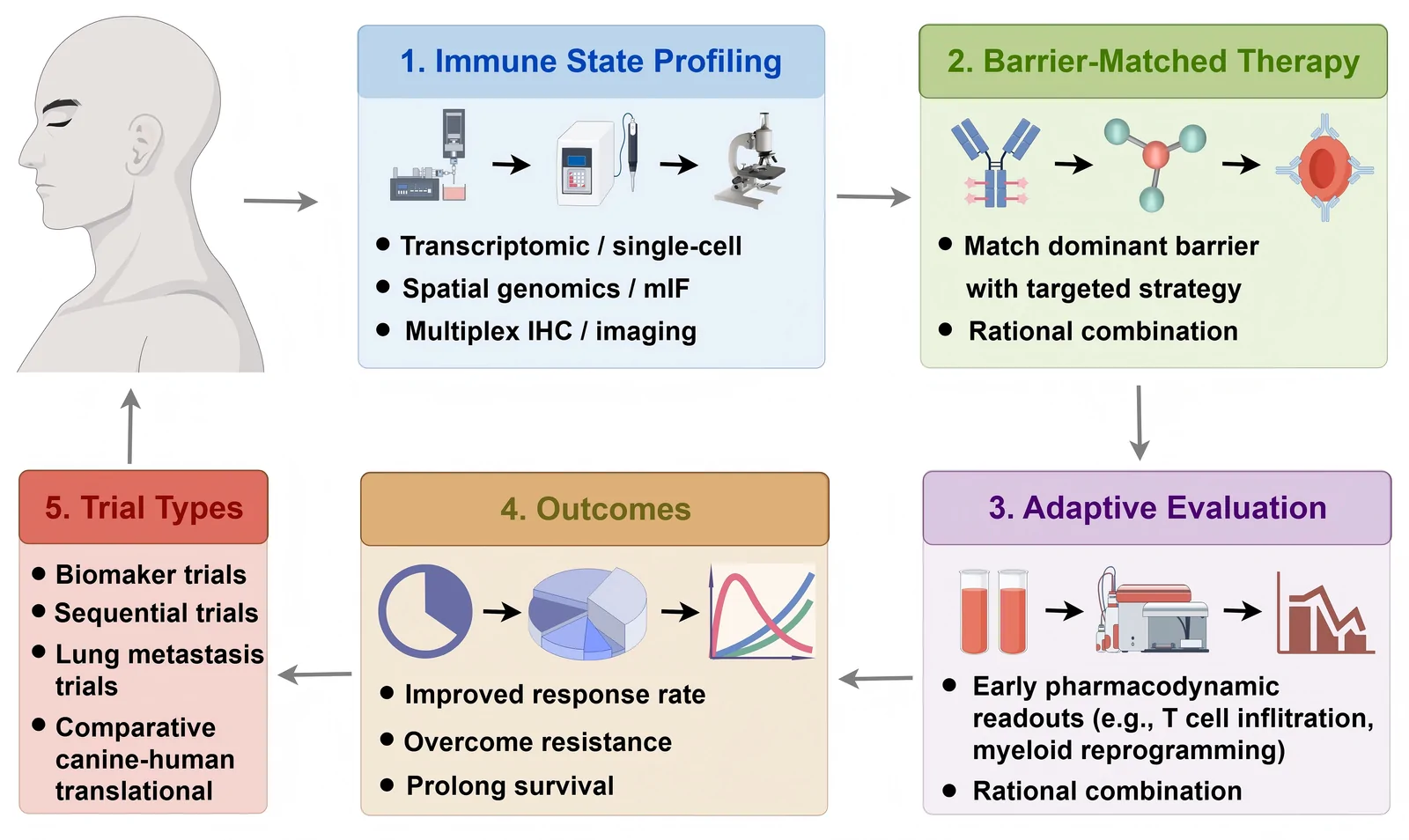
Immune state-guided trial framework for osteosarcoma immunotherapy. The proposed clinical framework begins with immune state profiling using transcriptomic, single-cell, spatial genomics, multiplex immunohistochemistry (IHC) or multiplex immunofluorescence (mIF) assays, ideally with metastatic-site-aware sampling. Treatment selection is then matched to the dominant barrier and evidence tier, followed by adaptive pharmacodynamic evaluation such as restored MHC-I expression, tumour-cell-adjacent T-cell entry or reduced suppressive myeloid activity. Outcomes should be interpreted as barrier reversal, response signals and resistance mapping; improved response rate or survival should be claimed only after prospective validation. The framework motivates biomarker-stratified, sequential/adaptive, lung-metastasis-focused and comparative canine-human translational trial designs.

The next generation of osteosarcoma immunotherapy trials should be guided by immune state diagnosis (76, 77). Rather than assigning treatment based solely on histology or stage, trials should first define the dominant immune barrier, including MHC-I loss or antigen-presentation defects, immune exclusion, myeloid dominance, fibroblast-rich stromal barriers, metabolic suppression, or lung niche–associated remodelling (**Figure 4**). These barriers can be evaluated using prespecified multimodal assays, including spatial immunofluorescence, spatial transcriptomics, single-cell RNA sequencing, bulk immune state classifiers, ctDNA-informed molecular stratification, and radiomic features associated with metastatic burden. Comprehensive multimodal profiling is unlikely to be feasible at every site or time point. A pragmatic tiered strategy could begin with a core panel based on routinely processed FFPE tissue: tumour-cell HLA-ABC and B2M to assess recognition (20, 35), CD8 density and tumour-cell proximity to assess immune access (18, 21), CD68 and CD163 to estimate macrophage abundance and polarization-associated patterns (15, 18, 21), and α-SMA or collagen-based fibrosis scoring to identify stromal exclusion (38, 40). These measurements should be interpreted together with anatomical site, treatment history, and metastatic status. Cases with discordant or indeterminate results could undergo second-tier testing using targeted RNA panels or multiplex immunofluorescence, whereas single-cell sequencing, spatial transcriptomics, and immune imaging could be reserved for nested translational studies. Serial ctDNA and peripheral immune profiling may complement tissue-based classification during follow-up but cannot substitute for spatial tissue assessment. Because routine MDSC phenotyping remains insufficiently standardized, it may initially be incorporated as an exploratory rather than core biomarker (78). Multicentre implementation will additionally require harmonized pre-analytical procedures, standardized staining and scoring protocols, external quality assurance, and either centralized testing or validated cross-site reproducibility (79). Cost and turnaround time should be incorporated as prespecified feasibility endpoints, with core FFPE-based results returned within clinically actionable timeframes and higher-cost spatial or single-cell assays reserved for unresolved cases or nested translational cohorts. This candidate minimal panel represents a feasible starting point but requires prospective analytical and clinical validation. In this context, adaptive trial designs may be particularly appropriate. Patients could be stratified through an integrated molecular–spatial screening platform, receive barrier-matched interventions, and then undergo pharmacodynamic reassessment to determine whether the targeted immune state has been modified. For example, in recognition-defective disease, evidence of restored MHC-I expression or improved tumour-cell recognition would serve as a readout of biological engagement.

Two hypothetical trial structures illustrate how this framework could be prospectively tested. Although neither design has been validated in osteosarcoma, biomarker-adaptive and response-directed neoadjuvant studies in other solid tumours provide methodological precedents for linking molecular stratification and early pharmacodynamic response to subsequent treatment assignment (80, 81). First, patients with recurrent osteosarcoma showing reduced tumour-cell MHC-I or B2M expression could enter a biomarker-enriched, adaptive phase Ib/II study incorporating a short antigen-presentation-restoration or immune-priming period (20, 23, 65). Baseline and on-treatment samples would be assessed for restoration of tumour-cell MHC-I expression, improved antigen-recognition capacity and increased tumour-cell-adjacent CD8^+^ T-cell density. Paired pretreatment and surgical specimens have similarly been used in neoadjuvant immunotherapy studies to evaluate pathological and immune responses before definitive surgery (82, 83). Patients demonstrating prespecified evidence of recognition-barrier reversal could then proceed to checkpoint blockade, whereas those without biological engagement could be redirected to an alternative barrier-matched cohort. This response-adaptive logic is conceptually analogous to the PRADO melanoma study, in which pathological response after neoadjuvant therapy informed subsequent surgical and adjuvant management, although the proposed immune-barrier readouts in osteosarcoma remain hypothetical (81). This design would test whether pharmacodynamic restoration of immune recognition precedes and predicts subsequent clinical response. Second, patients undergoing planned resection of pulmonary metastases could be enrolled in a window-of-opportunity study focused on immune-excluded or myeloid-dominant lesions. Baseline biopsy or archival metastatic tissue would be used to identify fibrocollagenous exclusion, CAF-rich architecture, peritumoral T-cell localization, or suppressive macrophage and MDSC enrichment. A short course of stromal- or myeloid-reprogramming therapy would then be administered before metastasectomy, allowing the resection specimen to be evaluated for increased penetration of effector T cells into tumour-cell regions, reduced suppressive myeloid activity and disruption of fibrosis-associated spatial barriers. Subsequent checkpoint or cellular therapy could be introduced only when predefined evidence of barrier reversal is observed. Such a design would provide a direct prospective test of whether spatial or myeloid reprogramming improves downstream immune responsiveness in lung-metastatic osteosarcoma.

In immune-excluded lesions, effective intervention would be reflected by T-cell penetration into tumour-cell regions. In myeloid-dominant disease, reduction in suppressive macrophage or MDSC activity would indicate pathway modulation (84, 85). Rather than serving as binary success or failure criteria, these readouts may help distinguish true target engagement from immune state mismatch or downstream resistance (**Figure 4**). This framework also highlights the importance of metastatic-site–specific evaluation. Lung metastases should not be treated as interchangeable with primary tumours. Future trials in recurrent or metastatic osteosarcoma should incorporate paired primary–metastatic sampling when feasible, spatial characterization of metastatic lesions, and pharmacodynamic endpoints capturing immune-cell localization across macrophage, fibroblast, and lymphocyte compartments. Collectively, this approach reframes immune coldness from a retrospective descriptor of therapeutic failure into a measurable, dynamic therapeutic state that can be prospectively interrogated and targeted (**Figure 4**).

Collectively, this framework proposes that future osteosarcoma trials should be structured around immune state–guided stratification rather than histology-based treatment assignment, with therapies selected according to the dominant tumour immune barrier. It further reframes therapeutic evaluation as barrier-specific pharmacodynamic modulation, enabling dynamic assessment of whether interventions successfully reprogram recognition, spatial, or myeloid immune dysfunction in a metastatic site–dependent context.

## Discussion

The principal value of the proposed framework lies in decomposing apparently similar immunotherapy-resistant phenotypes into mechanistically distinct barriers involving recognition, spatial access, myeloid–stromal suppression, and metastatic-site adaptation. Evidence from single-cell, spatial, and longitudinal studies supports substantial heterogeneity across treatment-naive, recurrent, and metastatic lesions, while also indicating that immune-cell abundance alone does not define productive immunity. The framework therefore extends beyond a binary hot-versus-cold classification by linking tumour-intrinsic, spatial, myeloid, and organ-specific features to testable therapeutic hypotheses. Its interpretation must nevertheless remain non-linear, because partially inflamed, exhausted, excluded, and myeloid-dominated states may coexist within the same patient or lesion.

Available human data do not support a uniform progression from immune-desert to increasingly suppressed states. In a longitudinal cohort of 30 samples from seven patients, metastatic lesions in some cases displayed stronger cytotoxic T-cell signatures, increased MAGEA expression, and even central T-cell infiltration compared with matched primary tumours (34). Variability in PD-1 and PD-L1 expression across diagnostic, resection, and metastatic samples further supports spatial and temporal heterogeneity (86). Single-cell studies also identify exhausted T-cell programs within tumours that retain measurable immune activity rather than representing complete immune absence (16). Together, these findings are more consistent with partially inflamed but functionally ineffective immune states than with a strictly linear immunological collapse. At the same time, this flexibility introduces interpretative limitations. A framework that permits retrospective assignment of heterogeneous lesions to multiple states risks reduced falsifiability if not constrained by prospective criteria. Additional limitations arise from the underlying evidence base, which is dominated by relatively small cohorts, publicly available transcriptomic datasets, computational deconvolution approaches, and prognostic signatures. Although immune-high and immune-low stratifications are reproducible across studies, their definitions remain dataset-dependent and lack standardized clinical thresholds (17, 87). Metabolic and hypoxia-associated signatures provide useful hypotheses regarding immune suppression, but inferred immune activity does not necessarily reflect spatial cell–cell interaction or therapeutic response *in vivo (*24). Even single-cell datasets are constrained by limited sampling depth, heterogeneity in treatment exposure, anatomical site variation, and analytical pipelines (15, 33). Accordingly, the proposed phases should be interpreted as operational constructs derived from existing literature rather than as validated biological stages emerging from a unified dataset.

Longitudinal validation remains the most important unresolved limitation. Among the human studies discussed in this Review, the serial immunogenomic analysis of seven patients provides the most direct evidence of immune state dynamics, but its limited size precludes the definition of reproducible trajectories or treatment-specific transition probabilities (34). Serial immunohistochemical analyses further indicate variability in checkpoint expression across specimens, although they do not resolve the multicellular mechanisms underlying these differences (86). Independent longitudinal whole-genome sequencing studies have shown that subclonal copy-number alterations can emerge and dominate at recurrence, reinforcing the need to consider tumour evolution and immune evolution in an integrated manner (88). Future studies will require systematically collected matched diagnostic, post-neoadjuvant, relapse, and metastatic specimens. In the absence of such sampling, apparent immune state differences may reflect inter-patient variability, anatomical sampling effects, tissue processing, decalcification artefacts, or clonal selection rather than true temporal transitions. Spatial evidence provides complementary but incomplete insight. Spatial transcriptomic analyses of paediatric pulmonary metastases have identified regionally distinct inflammatory, fibrocollagenous, and macrophage-enriched compartments, while multiplex immunofluorescence has linked specific cellular neighbourhoods containing PD-1–positive T cells and myeloid-derived suppressor cells with clinical outcome (18, 21). Integrated single-cell and spatial studies further implicate wound-healing and fibrotic programs surrounding disseminated tumour cells (38). However, these datasets remain limited in scale, rely on heterogeneous platforms and marker panels, and are largely derived from static surgical material. While they can define spatial adjacency, exclusion, and regional transcriptional programs, they do not by themselves establish directional cellular interactions or the temporal ordering of immune state transitions. Spatial interpretations also remain sensitive to section selection, tissue handling, and decalcification effects, all of which may influence RNA quality and apparent cellular architecture.

The evidence base is particularly fragmented for the lung metastatic niche. Preclinical studies collectively support a model in which tumour-derived secretomes and extracellular vesicles condition the lung microenvironment, recruit myeloid populations, activate fibroblasts, and promote fibrosis-like remodelling (29, 30, 37, 38). Additional mechanisms involving CCL2-dependent macrophage accumulation and GD2-mediated suppression of phagocytic activity further implicate myeloid dysfunction in pulmonary colonization (19, 25). However, much of this mechanistic evidence derives from cell-line systems, transplantation assays, or murine models, and may not fully recapitulate human disease. Human metastasectomy samples are inherently selected, represent established disease states, and do not capture the premetastatic or early seeding phases. Comparative canine studies provide an immunocompetent intermediate model and have revealed conserved tumour–microenvironment subtypes as well as pharmacodynamic responses to monocyte-targeted interventions (48, 49), but remain complementary rather than definitive. Most importantly, the proposed framework has not yet undergone prospective clinical validation. No study to date has stratified patients into recognition-defective, immune-excluded, myeloid-dominated, or metastatic niche–remodelled states and then evaluated whether barrier-matched interventions improve clinical outcomes. The modest activity of pembrolizumab in the osteosarcoma cohort of SARC028 is consistent with unresolved upstream barriers but does not identify them (4). Similarly, Alliance A091401 and subsequent checkpoint-based combination studies in heterogeneous sarcoma or bone sarcoma populations provide limited osteosarcoma-specific mechanistic resolution (5, 7, 8). Early HER2-directed CAR-T-cell and dendritic-cell vaccine studies further demonstrate feasibility without establishing predictive immune state–response relationships (59, 60). Taken together, these trials highlight not validation of the proposed framework, but rather the absence of prospective immune state stratification as a fundamental gap in current osteosarcoma immunotherapy.

Despite these constraints, the model may still be useful for clinical strategy design when treated as a hypothesis-generating framework rather than a prescriptive treatment algorithm. In this context, a recognition-defective lesion would be expected to require restoration of antigen presentation before checkpoint blockade can exert meaningful activity. Preclinical evidence is consistent with this sequence, including UBA2 inhibition and etoposide-mediated MHC-I upregulation (20, 65). In contrast, immune-desert or immune-excluded lesions may depend on T-cell recruitment or stromal remodelling, as suggested by MYC inhibition and cancer-associated fibroblast–targeted hydrogel approaches (40, 66). Myeloid-dominated or lung niche–adapted disease may require modulation of macrophage activity, phagocytosis checkpoints, or innate immune pathways prior to adaptive checkpoint intervention (39, 68). These observations collectively support the concept of sequential ecosystem reprogramming, although all supporting evidence remains preclinical. Importantly, the clinical implication is not that a fixed therapeutic sequence already exists, but rather that each intervention step should correspond to a measurable pharmacodynamic objective. Although sequential ecosystem reprogramming offers a mechanism-informed alternative to empiric combination therapy, its implementation may be limited by cumulative toxicity (83), treatment duration, sequencing logistics, biomarker turnaround, and the need for clinically stable patients. These constraints are particularly relevant in rapidly progressive metastatic disease. Early trials should therefore prioritize short biologically defined treatment windows (82, 83), agents with non-overlapping toxicities, rapid and parsimonious biomarker panels, and prespecified rules for treatment continuation, escalation, de-escalation, or switching (80, 81). The goal should be measurable barrier reversal with the fewest necessary interventions, while the feasibility, safety, treatment burden, and clinical value of this approach require prospective evaluation in osteosarcoma. Moving toward clinical validation will require progress across multiple levels. Analytical validation should establish reproducible assays and thresholds for key features such as MHC-I loss, tumour-adjacent T-cell density, suppressive myeloid architectures, and lung niche–associated fibrosis. While existing spatial datasets provide candidate readouts, cross-platform robustness and standardization remain unresolved (18, 21).

Biological validation will need to determine whether these immune states are stable across tumour regions and how they evolve under therapy. Evidence from paired and longitudinal sampling suggests that immune features and checkpoint expression can vary across disease sites and time points, underscoring the importance of serial tissue assessment where feasible (34, 86). Clinical validation will ultimately require embedding state assignment into prospective, multi-institutional trials with predefined biomarker readouts before and during treatment; particularly in paediatric osteosarcoma and pulmonary metastases, this will require parsimonious biomarker panels and carefully justified biopsy strategies rather than maximal tissue or assay collection. Practical longitudinal implementation will require a tiered monitoring strategy that reduces reliance on repeated tumour biopsies. Tissue obtained at diagnosis, clinically indicated resection, or relapse could serve as an anchoring reference, while serial blood- and imaging-based assessments provide complementary information between tissue time points. Serial ctDNA analysis may track tumour burden, post-operative minimal residual disease, and relapse risk (89), but cannot resolve spatial immune exclusion or myeloid–stromal architecture. Circulating immune profiling, including multiparameter flow cytometry, soluble mediators, and T-cell receptor repertoire analysis, may capture systemic pharmacodynamic changes, although concordance with intratumoral states requires validation (90). Radiomic analysis may enable repeatable lesion-level assessment, but osteosarcoma studies have mainly focused on retrospective prediction of chemotherapy response rather than validated immune-state monitoring (91). Emerging CD8- or granzyme B-targeted PET may further visualize effector-cell distribution or activity (92, 93), although osteosarcoma-specific validation, tracer availability, radiation exposure, and paediatric feasibility remain important limitations. A pragmatic strategy could therefore combine tissue-based reference measurements with serial ctDNA, peripheral immune profiling, standardized radiomics, and immune imaging in nested feasibility cohorts.

To remain scientifically useful, the framework must generate explicit predictions that can be prospectively tested and, if necessary, refuted. First, the four proposed barriers should be reproducibly assigned using prespecified molecular and spatial criteria across independent cohorts and analytical platforms. Second, reversal of the dominant barrier should precede or accompany therapeutic response. Third, barrier-matched interventions should produce greater pharmacodynamic and clinical benefit than mechanistically mismatched strategies in comparable disease contexts. Fourth, pulmonary metastases should display reproducible organ-specific immune features that cannot be explained solely by treatment exposure, tumour burden, sampling, or technical variation. The framework would require revision if the proposed states cannot be assigned reproducibly, if barrier reversal is uncoupled from therapeutic response, if matched strategies do not outperform mismatched treatment, or if independent datasets consistently support fewer, different, or additional biologically distinct states. In its current form, the evolutionary immune coldness model should therefore be regarded as a structured, hypothesis-generating framework rather than a validated clinical classifier or natural history model (4, 18, 34). Its primary contribution is to render therapeutic resistance mechanistically decomposable and experimentally testable. Its future relevance will depend on whether longitudinal, spatial, and biomarker-driven studies can translate this conceptual structure into reproducible immune state definitions and clinically actionable stratification.

## Conclusions

Osteosarcoma should not be labelled as immune-cold solely on the basis of limited responses to checkpoint blockade. A more informative view is that it encompasses a spectrum of non-productive immune states that vary with tumour stage, prior treatment exposure, and metastatic site. Treating these heterogeneous states as a single phenotype risks obscuring the biological diversity underlying immunotherapy resistance. This Review proposes a conceptual framework in which recognition failure, immune exclusion, myeloid dominance, and metastatic immune-ecosystem formation are considered interconnected but non-sequential barriers that collectively produce non-productive immunity as a downstream clinical outcome. From a therapeutic perspective, this implies that effective immunotherapy will depend on identifying the dominant immune barrier, rather than applying uniform treatment strategies. In different contexts, checkpoint blockade, macrophage-targeting approaches, STING or oncolytic therapies, metabolic interventions, fibroblast modulation, or cellular therapies may each be relevant, but none is expected to be broadly effective across all immune states. Ultimately, a mechanism-oriented and evolution-aware perspective may help shift immune coldness from a descriptive label toward a measurable and testable framework for therapeutic decision-making, enabling more context-specific treatment strategies in osteosarcoma.
